# The HIPPO Transducer YAP and Its Targets CTGF and Cyr61 Drive a Paracrine Signalling in Cold Atmospheric Plasma-Mediated Wound Healing

**DOI:** 10.1155/2020/4910280

**Published:** 2020-02-13

**Authors:** Debarati Shome, Thomas von Woedtke, Katharina Riedel, Kai Masur

**Affiliations:** ^1^Leibniz Institute for Plasma Science and Technology, A Member of the Leibniz Research Alliance Leibniz Health Technology, Felix-Hausdorff-Straße 2, Greifswald 17489, Germany; ^2^Institute for Microbiology, University of Greifswald, Felix-Hausdorff-Straße 8, Greifswald 17489, Germany

## Abstract

Reactive species play a pivotal role in orchestrating wound healing responses. They act as secondary messengers and drive redox-signalling pathways that are involved in the homeostatic, inflammatory, proliferative, and remodelling phases of wound healing. The application of Cold Atmospheric Plasma (CAP) to the wound site produces a profusion of short- and long-lived reactive species that have been demonstrated to be effective in promoting wound healing; however, knowledge of the mechanisms underlying CAP-mediated wound healing remains scarce. To address this, an *in vitro* coculture model was used to study the effects of CAP on wound healing and on paracrine crosstalk between dermal keratinocytes and fibroblasts. Using this coculture model, we observed a stimulatory effect on the migration ability of HaCaT cells that were cocultured with dermal fibroblasts. Additionally, CAP treatment resulted in an upregulation of the HIPPO transcription factor YAP in HaCaTs and fibroblasts. Downstream effectors of the HIPPO signalling pathway (CTGF and Cyr61) were also upregulated in dermal fibroblasts, and the administration of antioxidants could inhibit CAP-mediated wound healing and abrogate the gene expression of the HIPPO downstream effectors. Interestingly, we observed that HaCaT cells exhibited an improved cell migration rate when incubated with CAP-treated fibroblast-conditioned media compared to that observed after incubation with untreated media. An induction of CTGF and Cyr61 secretion was also observed upon CAP treatment in the fibroblast-conditioned media. Finally, exposure to recombinant CTGF and Cyr61 could also significantly improve HaCaT cell migration. In summary, our results validated that CAP activates a regenerative signalling pathway at the onset of wound healing. Additionally, CAP also stimulated a reciprocal communication between dermal fibroblasts and keratinocytes, resulting in improved keratinocyte wound healing in coculture.

## 1. Introduction

Cold Atmospheric Plasma (CAP) is an emerging tool of biomedical and clinical importance that controls cellular processes such as wound healing, immunomodulation, cell death, and cancer. CAP is a partially ionized molecular or noble gas or gas mixture that generates a plethora of reactive oxygen and nitrogen species (ROS/RNS), radiation (ultraviolet (UV), visible, and infrared), and small chemical entities such as neutral molecules, ions, electrons, and exited atoms under atmospheric pressure and ambient temperature [1]. The short- and long-lived ROS and RNS produced by CAP are involved in redox signalling *in vitro* and *in vivo.* Wound healing involves a coordinated infiltration of dermal cells that is followed by infiltration of immune cells, extracellular matrix deposition, and reepithelization over distinct yet interdependent phases that are regulated by a large number of transcription factors or transcriptional events [2–5]. ROS are critical secondary messengers that orchestrate wound healing processes by regulating the recruitment of immune cells, angiogenesis, and optimal perfusion of blood at the wound site [6]. Recent reports also indicate that CAP supports wound healing by influencing cell viability [7, 8], proliferation [9], migration [10], inflammation [11], dermal regeneration, and reepithelization [12]. Currently, only a small number of studies have examined the cellular and molecular effects of CAP-mediated wound healing [1, 13, 14] and the mechanisms underlying CAP-mediated wound healing in the context of regenerative pathways require further investigation.

The HIPPO signalling pathway was initially identified in *Drosophila melanogaster*, and this pathway regulates cellular proliferation, organ size, and survival and is highly conserved in mammals [15–17]. The core HIPPO pathway in vertebrates consists of a kinase cascade where one of the major transcriptional coactivators is Yes-associated protein (YAP) [18, 19]. Nonphosphorylated YAP translocates to the nucleus and induces target gene transcription by interacting with the transcription factors TEA domain family member proteins (TEAD 1-4) [20]. In particular, YAP-mediated target gene transcription depends upon its cellular localization (cytoplasmic or nuclear) [21–24].

The HIPPO signalling pathway is of paramount importance in the oxidative stress response and wound healing. YAP is involved in redox homeostasis regulation, and its deletion can lead to redox sensitivity [22]. Certain YAP-targeting genes encode secretory proteins and may possess regenerative potential [25]. The downstream target genes of YAP that are of primary interest in this study are Connective Tissue Growth Factor (CTGF) and Cysteine-rich angiogenic protein 61 (Cyr61), members of CCN family proteins. According to literature, YAP moves to the nucleus and interacts with TEAD to promote the transcription of these matricellular proteins that are involved in cell adhesion and migration [26, 27]. These extracellular matrix-associated signalling proteins are structurally related heparin-binding proteins and are synthesized during cutaneous wound healing in dermal fibroblasts [28]. Due to its ability to modulate the activities of several growth factors and cytokines, CTGF possesses the potential to regulate diverse biological processes including cell adhesion, migration [29], proliferation [30], angiogenesis [31], and early wound healing and repair [32]. A physiological role of Cyr61 in regulating inflammation, adhesion, angiogenesis, proliferation, matrix remodelling, and cell-ECM interaction during wound repair has also been identified [26, 28, 33, 34]. Oxidative stress plays a role in the alteration of CTGF expression in fibroblasts [35]. Qin et al. found that Cyr61 expression is induced by oxidative stress and that a positive feedback loop continues to generate ROS and upregulate Cyr61 in fibroblasts [36]. Taken together, these reports strongly suggest the presence of a redox-induced HIPPO signalling axis in wound healing that is complex and poorly understood.

In this study, for the first time, we demonstrate the activation of the YAP-CTGF-Cyr61 axis in response to CAP treatment in a well-characterized human epithelial cell line (HaCaT) and in a human nonneonatal skin fibroblast cell line (GM00637). CAP-mediated acceleration of wound healing indicates the presence of a paracrine signalling pathway that is driven by the extracellular matrix-associated signalling proteins CTGF and Cyr61 in a coculture model. The activation of organ survival and regenerative pathways further establishes the usefulness of CAP in redox-mediated wound management and repair.

## 2. Materials and Methods

### 2.1. Cell Culture

Human skin keratinocytes (HaCaT) and the immortalized skin fibroblast cell line GM00637 (GM Fbs) were cultured in Roswell Park Memorial Institute 1640 cell culture medium (RPMI 1640, Invitrogen) supplemented with 10% fetal calf serum (FCS, Invitrogen), 0.1 mg/ml penicillin/streptomycin, and 2 mM L-glutamine (PAN Biotech, Germany). For 2D scratch assays, the indicated number of cells (either in mono- or coculture), as per experimental requirements, was incubated in 96-well image lock culture plates (Essen BioScience) in a starved cell culture medium (supplemented with 1% FCS) 16-18 hr prior to their experimental use.

#### 2.1.1. Coculture

Direct coculture of HaCaTs and GM Fbs was used for cell migration assays and metabolic assays in 96-well image lock culture plates (Essen BioScience) or normal 96-well plates (Sarstedt), respectively. For coculture experiments, GM Fbs were seeded first into a 96-well plate, and once they adhered to the plate, the indicated number of HaCaTs was seeded on top of the fibroblasts in a manner that was dependent upon experimental type. The exact cell numbers are described in the respective method sections. The cell numbers (in mono- or coculture) and the experimental type are also summarized in [Supplementary-material supplementary-material-1].

### 2.2. Harvest of GM-Fb-Conditioned Medium (GM-Fb-CM)

To harvest the conditioned medium (CM), GM Fbs (0.1 × 10^6^/well) were seeded onto a 24-well plate in complete RPMI 1640 (supplemented with 10% FCS) until the cells were approximately 80% confluent. The medium was then replaced with 1% FCS medium for 16-18 hr. Next, fibroblasts were incubated with untreated or CAP-treated medium for another 18 hr. Following the incubation, conditioned medium was collected. The CM was centrifuged at 2000 rpm for 5 min, filtered through a 0.22 *μ*m syringe filter, and kept at 4°C for immediate use. For longer storage, the medium was stored at −80°C.

### 2.3. CAP Treatment

CAP treatment was performed using an atmospheric pressure argon plasma jet kINPen MED (neoplas tools, Greifswald, Germany). The electrical safety of the kINPen was certified and complies with EU standards [37]. This is the first jet worldwide to be accredited as a medical device class IIa according to European Council Directive 93/42/EEC for use in wound treatment, and this device lacks mutagenic and genotoxic effects [37–39]. The jet was operated at a voltage of 2-6 kVpp and a frequency of 1 MHz. The gas flow rate was set at 5 SLM (standard litres per minute) to treat 5 ml of RPMI 1640 supplemented with 1% FCS in a 60 mm dish for 10, 30, and 60 s. CAP-treated media were immediately used to treat the cells for further experiments. Cells incubated with untreated RPMI medium served as negative controls for all experiments.

### 2.4. Metabolic Activity Assay

To determine metabolic activity, 2.1 × 10^4^ cells (in monoculture) were seeded into a 96-well plate (Sarstedt) 24 hours prior to the start of the experiment. For coculture, 0.7 × 10^4^ GM Fbs and 1.4 × 10^4^ HaCaTs were seeded onto the plate. After incubation with CAP-treated media for 3 and 24 hr, the wells were loaded with 100 *μ*M of resazurin (Alfa Aesar) that is transformed to fluorescent resorufin by metabolically active cells. The plate was incubated for 2 h at 37°C, and fluorescence was measured using a Tecan plate reader (Infinite M200 Pro) at *λ*_ex_ 535 nm and *λ*_em_ 590 nm. The fluorescence values were normalized to those of the untreated controls.

### 2.5. Cell Migration Assay

The cell migratory behaviour of HaCaTs and GM Fbs was assayed using a cell migration assay that incorporated an Essen BioScience wound maker. For this assay, separate monocultures of HaCaT and GM Fbs and coculture of HaCaT and GM Fbs were used. For the monocultures, either 6 × 10^4^ HaCaTs/well or GM Fbs/well were seeded into Essen BioScience 96-well image lock plates. For the coculture, 2 × 10^4^ GM Fbs/well were seeded into image lock plates and incubated until the fibroblasts attached to the plates, and then 4 × 10^4^ HaCaTs/well were seeded onto the top of the fibroblasts at a ratio of 1 : 2 fibroblasts: keratinocytes within the coculture. Several other ratios (1 : 3, 1 : 5) of HaCaTs and GM Fbs were also tested for the cell migration assays (data not shown), but in further experiments, a 1 : 2 ratio of HaCaT and GM Fbs was used. After complete adherence of HaCaTs and GM-Fbs in mono- and cocultures, the cells were starved using 1% FCS for 16-18 hr prior to the experiment. The Essen BioScience wound maker was used to create scratches, and the cells were then incubated with either 10 or 30 s plasma-treated medium or untreated medium as indicated.

For other experiments, recombinant CTGF (10-50 ng/ml) and Cyr61 (100-1000 ng/ml) (PeproTech GmBH) and GM-Fb-CMs were used on HaCaT monocultures seeded at a density of 4 × 10^4^ cells/well.

The cell migration rate was monitored using an IncuCyte S3 live cell analysis system for up to 24 hr. The relative wound density was measured with the in-built software of the IncuCyte S3 2018A. The relative wound density (%) is the ratio of the occupied area of the initially scratched area with respect to the total scratched area. The graphs are plotted as a function of relative wound density (%) in *y*-axis in comparison to the time at the *x*-axis.

#### 2.5.1. mRNA Expression Analysis

Quantitative real-time PCR was performed using a 96-well LightCycler 480 qPCR system according to the manufacturer's protocol (Roche Diagnostics, Germany). Briefly, 0.5 × 10^6^ HaCaTs or GM Fbs were seeded into a 6-well plate, and once the monocultures reached confluence, the cells were starved for 16-18 hours, scratches were made using a 10 *μ*l pipette tip, and the cells were subsequently incubated with CAP-treated medium or untreated control medium. Total RNA was isolated with an RNA mini kit (Bio & Sell, Germany) after 3, 6, 18, and 24 hr. 1 *μ*g of RNA was transcribed to cDNA using a High Capacity cDNA Reverse Transcription Kit (Thermo Fisher Scientific), and qPCR was performed in triplicate with SYBR Green I Master mix (Roche Diagnostics, Germany) specific for the genes YAP, CTGF, and Cyr61. Primer sequences are provided in [Supplementary-material supplementary-material-1]. The housekeeping gene *β*-actin was used as the internal control for normalization. Gene expression analysis was performed using the *ΔΔ*CT method [40]. The final fold change in gene expression upon plasma treatment was determined according to the ratio of expression in the respective sample corresponding to the control.

### 2.6. Immunoblotting

HaCaTs and GM Fbs were seeded at a density of 0.5 × 10^6^ cells in a 6-well plate. After the cells reached confluency, they were serum starved for 16-18 hours, scratches were created using a 10 *μ*l pipette tip, and the cells were subsequently incubated with CAP-treated medium or untreated medium. Cells were harvested into ice-cold PBS after 3, 6, and 24 hr. Next, the samples were lysed in RIPA buffer that was supplemented with protease and phosphatase inhibitors (Roche) for 20 min on ice. Lysates were then centrifuged at 15000 g for 15 min at 4°C, and total extracted protein was quantified using the DC protein assay (Bio-Rad). Total protein (25 *μ*g) was resolved by 10% SDS-PAGE (Bio-Rad) and then transferred and blotted on PVDF membranes. The membranes were probed with antitotal YAP (Santa Cruz Biotechnology), phospho-YAP (CST), CTGF (Novex Biotechnology), Cyr61 (CST), and *β*-actin (CST) primary antibodies, and this was followed by incubation with secondary horse radish peroxide- (HRP-) coupled antibodies (CST) and subsequent detection of the bands using chemiluminescence (Applied Biosystems). Band intensities were quantified with Image Studio Lite (version 5.2), normalized to housekeeping *β*-actin, and expressed as a fold change compared to corresponding untreated control.

### 2.7. N-Acetyl Cysteine (NAC) Treatment

A 100 mM stock solution was prepared by dissolving NAC powder (Sigma-Aldrich) into 1x sterile PBS. This solution was sterilized using a 0.2 *μ*M sterile membrane filter, and aliquots of the stock solution were stored at −20°C. HaCaTs and GM Fbs were incubated with 2.5 mM NAC in the presence or absence of CAP-treated media for the designated times and then harvested for further experiments.

### 2.8. Enzyme-Linked Immunosorbent Assay (ELISA)

GM-Fb-CM was collected 18 hours after incubation with 10 and 30 s of CAP-treated or untreated medium and analysed using enzyme-linked immunosorbent assay (ELISA). The concentrations of secreted CTGF (PeproTech GmBH, catalogue no: 900-K317) and Cyr61 (R&D systems, catalogue no: DCYR10) were quantitatively determined according to the manufacturer's protocol. ELISA values were measured in duplicate from two different biological experiments for each sample to minimize the intra- and interassay variability.

### 2.9. Statistical Analysis

Graphics and statistics were performed using prism 8.02 (GraphPad PRISM software, San Diego, USA). Mean and standard deviation were calculated and analysed according to unpaired *t*-test with Welch's correction and ordinary one-way or 2-way analysis of variance (ANOVA). A *p* value < 0.05 was considered statistically significant (^∗^*p* ≤ 0.05, ^∗∗^*p* ≤ 0.01, ^∗∗∗^*p* ≤ 0.001, and ^∗∗∗∗^*p* ≤ 0.0001).

## 3. Results

### 3.1. Influence of CAP on Metabolic Activity in Mono- and Coculture

**Figure 1:**
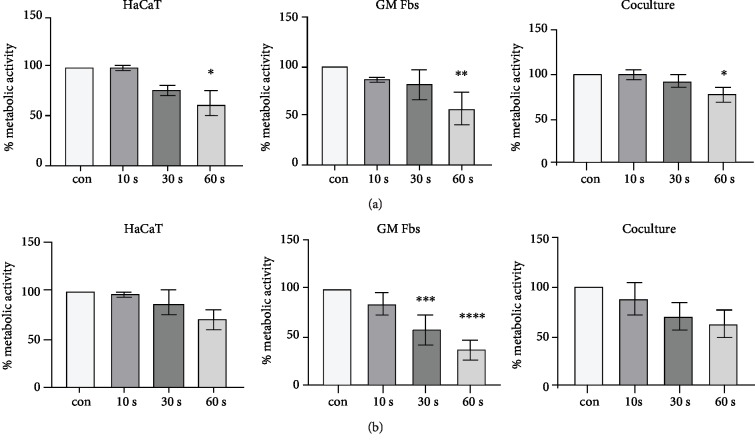
Influence of CAP on the metabolic activity in mono- and coculture: the metabolic activity of HaCaTs, GM Fbs, and coculture after 3 hr in response to 10, 30, and 60 s of CAP treatments. Untreated RPMI served as control (a). Metabolic activity of the same cell types after 24 hours of CAP treatment (b). Data are presented as mean ± SD of three independent experiments performed in triplicate. Statistical comparisons were performed using 1-way ANOVA.

There are compelling evidences within the literature indicating that among all the short- and long-lived ROS produced by CAP, H_2_O_2_ is one of the most stable oxidants produced by kINPen MED [41]. Based on this, we quantified the H_2_O_2_ concentration (in *μ*M) and found that there is a linear increase in H_2_O_2_ concentration according to treatment time ([Supplementary-material supplementary-material-1]). CAP did not significantly alter metabolic activity after 10 and 30 s of treatment; however, a decrease was observed in response to a 60 s of treatment after 3 hr of incubation in all cell types (Figure 1(a)). Further significant reduction was not observed in the metabolic activity of HaCaTs and cocultured cells after 24 hr, but a 30 and 60 s treatment resulted in a significant reduction in metabolic activity in GM Fbs after 24 hr (Figure 1(b)). Based on this, we chose 10 and 30 s treatments for all subsequent experiments.

### 3.2. CAP Accelerates Cell Migration in Coculture

**Figure 2:**
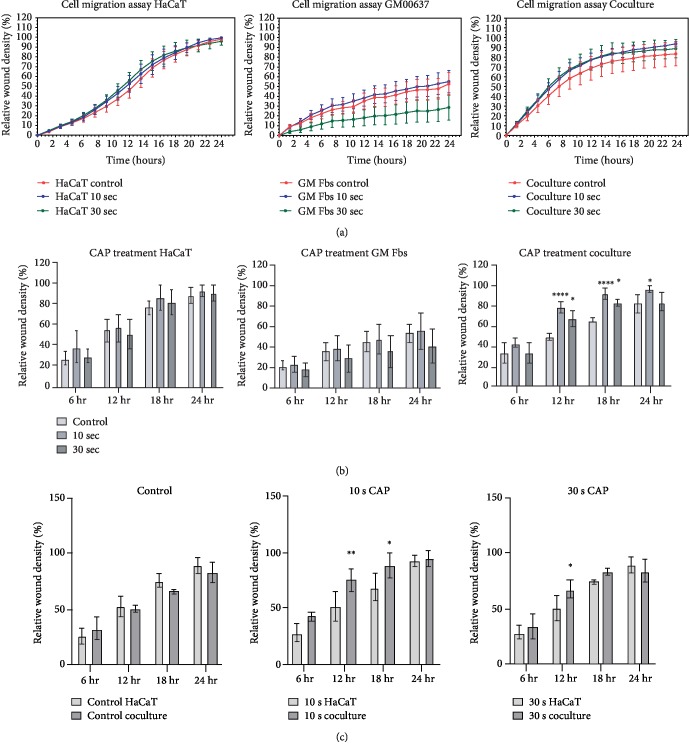
CAP accelerates cell migration in coculture: (a) depicts the representative time course of wound closure in untreated or CAP-treated HaCaTs, GM Fbs, and coculture, respectively, (upper row) up to 24 h. (b) shows the summation of relative wound density of untreated and CAP-treated HaCaTs, GM Fbs, and coculture, respectively, (middle row) at specific time points (6, 12, 18, and 24 h). (c) details the comparison of relative wound density between untreated and CAP-treated HaCaTs and coculture at 6, 12, 18, and 24 hr (lower row). Data are presented as mean ± SD of three independent experiments performed in triplicate. Statistical comparisons were performed using 2-way ANOVA.

Migration and proliferation of keratinocytes and fibroblasts are key biological processes that occur during various steps of wound healing, including wound repair and reepithelization. To exclusively determine cell migration, all cell types were starved prior to performing the scratch assay. Based on the results, CAP did not significantly alter relative wound density in either of the cell lines after 10 and 30 s of treatments (Figures 2(a) and 2(b), first and second column); however, CAP significantly accelerated relative wound density in coculture after 12 hours (Figures 2(a) and 2(b), third column). Wound closure images of both the monocultures and the coculture are provided in the supplementary figures ([Supplementary-material supplementary-material-1], [Supplementary-material supplementary-material-1], and [Supplementary-material supplementary-material-1]). We also compared the relative wound density between HaCaTs alone and those cocultured with fibroblasts, and we observed that after CAP treatment, relative wound density was significantly increased in coculture compared to that observed in HaCaT monoculture (Figure 2(c)). These results indicated that the CAP-mediated effect on wound closure may differ between mono- and coculture. However, as coculture is more closely related to skin models due to the inclusion of both keratinocytes and fibroblasts, we assessed the signalling pathways that were active in each cell type to determine the stimulatory effect of CAP on coculture migration.

### 3.3. CAP Accelerates Migration through the Activation of the YAP-CTGF-Cyr61 Axis

**Figure 3:**
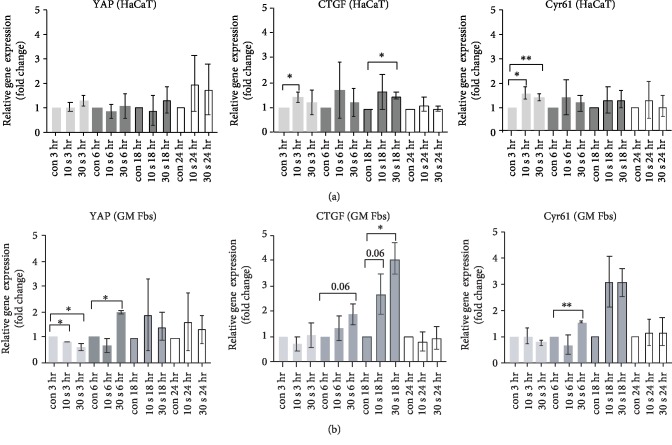
mRNA expression of HIPPO signalling effectors after CAP treatment: mRNA expression of YAP, CTGF, and Cyr61 in HaCaTs (a) and GM Fbs (b) measured 3, 6, 18, and 24 hr after CAP treatment by qPCR and normalized to relative gene expression (*ΔΔ*CT values on a log2 scale). The *x*-axis represents CAP treatment time and incubation time after plasma treatment. Data are represented as mean ± SD of either three (a) or four (b) independent experiments. Statistical analysis was performed using unpaired *t*-test with Welch's correction for multiple comparisons, along with normalization to the untreated control.

Quantitative real-time PCR revealed that CAP significantly stimulated YAP mRNA expression at 6 hr in GM Fbs (Figure 3(b)). CAP also tends to increase YAP at late time points in GM Fbs (18 hr) and HaCaTs (at 24 hr) (Figures 3(a) and 3(b)), and CAP minimally induced CTGF and Cyr61 in HaCaTs at 3 hr (Figure 3(a)). However, CAP increased CTGF and Cyr61 gene expression to more than threefold in GM Fbs, and this peaked 18 hr after treatment and then declined to near baseline by 24 hr (Figure 3(b)).

**Figure 4:**
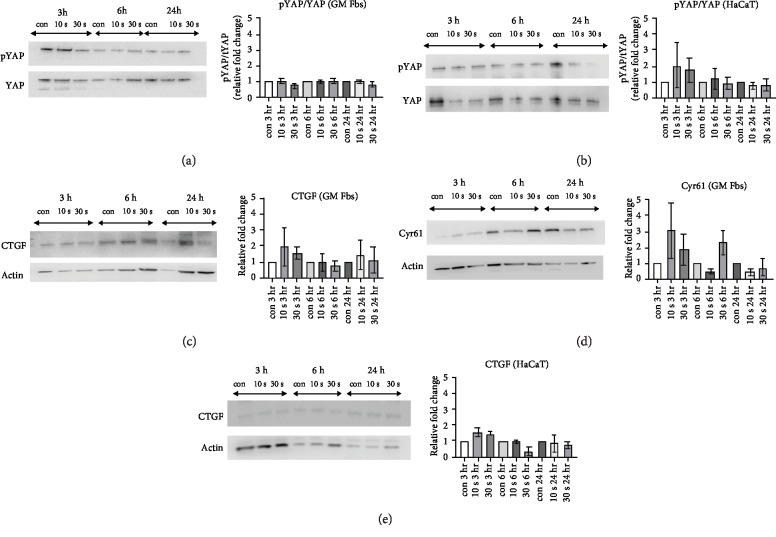
Effect of CAP on the HIPPO signalling pathway: the relative phosphorylation level of YAP is shown in GM Fbs (a) and HaCaT cells (b). CTGF and Cyr61 protein expression was enhanced after 10 and 30 s of CAP treatment in GM Fbs (c, d). CTGF expression slightly increased after 10 and 30 s of CAP treatments in HaCaT cells after approximately 3 hours (e). Representative blots are shown. The *x*-axis represents CAP treatment time and incubation time after plasma treatment. Data are represented as mean ± SD of at least three independent experiments. Statistical analysis was performed using unpaired *t*-test with Welch's correction for multiple comparisons, along with normalization to the untreated control.

Under serum-starved conditions, the ratio of phosphorylated YAP compared to total YAP (pYAP/YAP) protein was not significantly altered in GM Fbs (Figure 4(a)); however, there was a tendency for increased phosphorylation in HaCaT cells (Figure 4(b)). We also observed an increase in total cellular CTGF (3 hr) and Cyr61 protein (3 hr and 6 hr) levels after CAP treatment in GM Fbs (Figures 4(c) and 4(d)); however, unlike in GM Fbs, we could only detect a minor increase of CTGF in HaCaT cells upon CAP treatment (Figure 4(e)) and Cyr61 protein expression was completely undetectable (data not shown).

### 3.4. Effect of NAC on Metabolic Activity and Cell Migration

**Figure 5:**
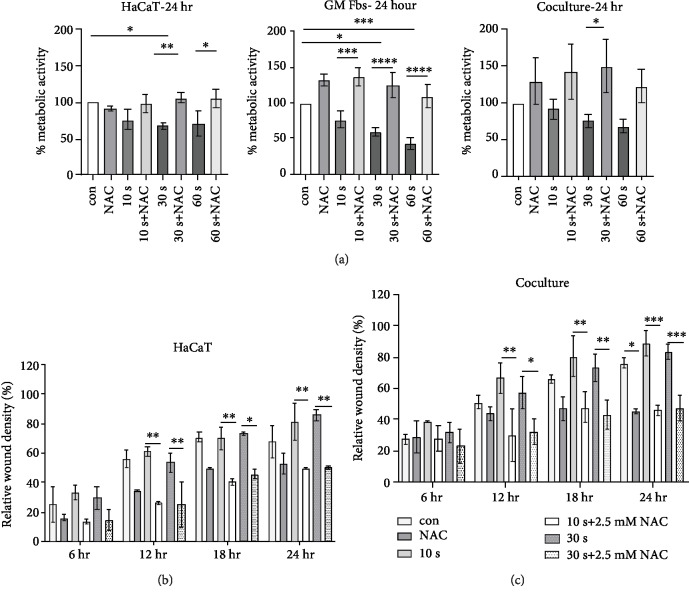
Effect of NAC on CAP-mediated wound healing and metabolic activity: metabolic activity of HaCaTs, GM Fbs, and coculture after 24 hr incubation with 2.5 mM NAC alone or in combination with CAP-treated media (a). Relative wound density of HaCaTs after treatment with NAC alone or in combination with CAP-treated media at 6, 12, 18, and 24 hr (b). Relative wound density of coculture after treatment with NAC alone or in combination with CAP-treated media at 6, 12, 18, and 24 hr (c). Data are presented as mean ± SD from at least three independent experiments performed in triplicate (a–c). Statistical analysis was performed using either unpaired *t*-test with Welch's correction (a) or 2-way ANOVA (b, c).

NAC is a well-known inhibitor of reactive species, and to determine the effect of CAP-produced ROS on cell signalling, cells were incubated with 2.5 mM NAC alone or in combination with differential CAP-treated media for 24 hr. After 24 hr, NAC alone did not significantly alter the metabolic activity of the cells. In HaCaTs, NAC significantly counteracted the 30 and 60 s CAP treatment effects. In GM Fbs, NAC counteracted 10, 30, and 60 s CAP treatments and increased the metabolic activity. In coculture, NAC significantly counteracted the effects of 30 s of CAP treatment and increased the metabolic activity of these cells (Figure 5(a)). Although metabolic activity was restored, we still sought to determine if CAP-induced wound healing signalling is affected after NAC administration. Therefore, cell migration assays were performed using HaCaT monoculture and coculture. The cells were incubated with CAP-treated media with or without 2.5 mM NAC. Interestingly, we observed that NAC alone did not improve HaCaT cell migration. CAP treatment actually resulted in a modest acceleration in HaCaT cell migration that was significantly reduced after NAC administration (Figure 5(b)). Similar results were obtained in coculture experiments, where NAC reduced CAP-mediated cell migration to ~40-50%. (Figure 5(c)).

### 3.5. Effect of NAC on YAP-CTGF-Cyr61 Axis

**Figure 6:**
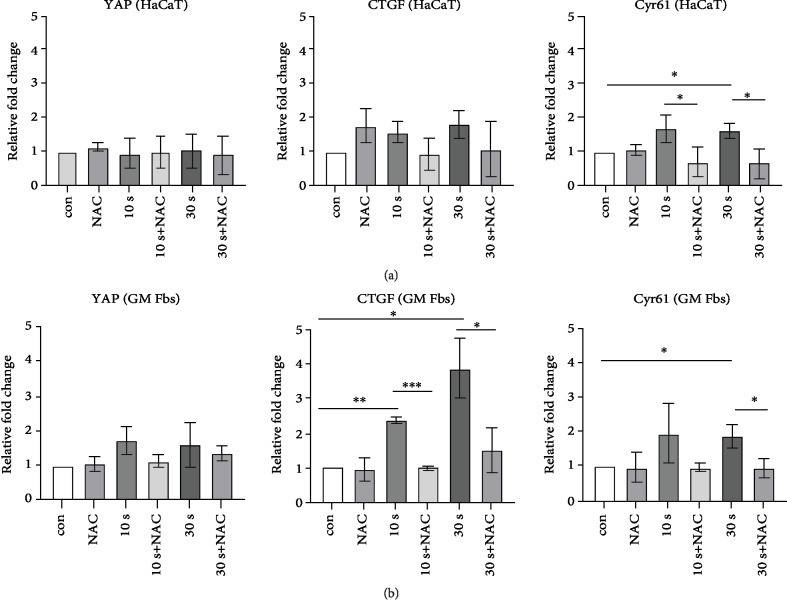
Effect of NAC on the YAP-CTGF-Cyr61 signalling cascade: mRNA expression of YAP, CTGF, and Cyr61 in HaCaTs (a) and GM Fbs (b) measured 18 hr after CAP treatment by qPCR and normalized to relative gene expression (*ΔΔ*CT values on a log2 scale). Data are presented as mean ± SD from at least three independent experiments, and statistical analysis was performed using unpaired *t*-test with Welch's correction.

We previously observed a CAP-mediated activation of the YAP-CTGF-Cyr61 signalling cascade. To confirm that the upregulation of this signalling is mediated by CAP-induced reactive species, total RNA was isolated from HaCaTs and GM Fbs after incubation with NAC alone or with CAP-treated media. An 18-hour incubation time in the presence of CAP-treated media was chosen for gene expression analysis. NAC in combination with 10 and 30 s CAP-treated media significantly reduced the expression of CTGF (~2.4 fold) and Cyr61 (~1.9 fold) in GM Fbs. YAP expression was also reduced; however, NAC alone did not significantly impact these signalling molecules (Figure 6(b)). Similar results were obtained in HaCaT cells that revealed that NAC significantly reduced the CAP-mediated Cyr61 increase; however, YAP expression remained unaltered and CTGF expression was slightly but not significantly reduced (Figure 6(a)).

### 3.6. CAP Promotes Migration in Coculture through Secreted CTGF and Cyr61

**Figure 7:**
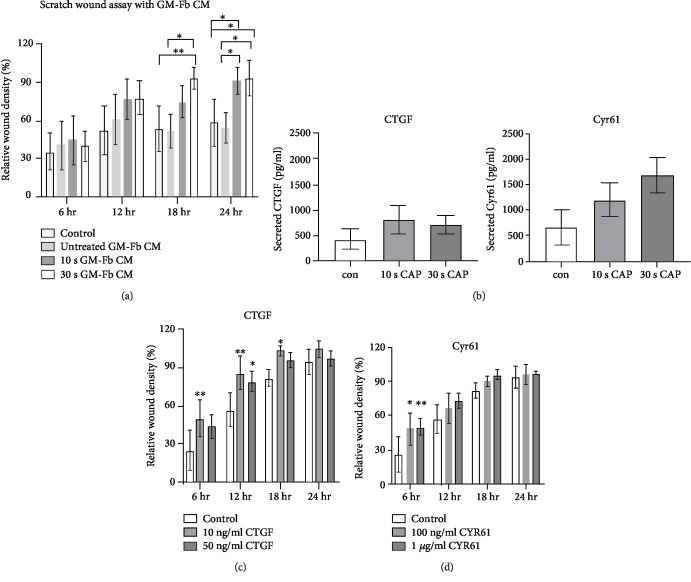
Effect of CAP on paracrine signalling between keratinocytes and fibroblasts: HaCaT cells were incubated with conditioned media harvested from untreated or 10 and 30 s CAP-treated GM Fbs. Relative wound density of HaCaT cells was monitored after 6, 12, 18, and 24 hr (a). Secreted CTGF and Cyr61 (pg/ml) was analysed by ELISA (b). Relative wound density in HaCaT cells was monitored after treatment with 10 and 50 ng/ml CTGF and 100 ng/ml and 1 *μ*g/ml Cyr61 (c, d). Data are presented as mean ± SD from three (a, c, d) or two (b) independent experiments, and statistical analysis was performed using 1- (b) or 2-way ANOVA (a, c, d).

CAP-induced migration was higher in HaCaTs cocultured with GM Fbs than in monoculture alone (see Figure 2), indicating that CAP regulates the interaction between these cells in coculture. We assumed that the major regulatory factors are CTGF and Cyr61 in coculture based on these data. We performed a cell migration assay to determine the migratory behaviour of HaCaTs grown in media conditioned from untreated or CAP-treated GM Fbs. Conditioned medium harvested from CAP-treated GM Fbs at 18 hr significantly accelerated HaCaT cell migration compared to conditioned medium from untreated GM Fbs and normal medium (Figure 7(a)). We also analysed the GM-Fb-CM by ELISA, and we observed an increase in secreted CTGF and Cyr61 after CAP treatment (Figure 7(b)).

Recombinant CTGF and Cyr61 were also used to assess cell migration. HaCaT monoculture was treated with 10 and 50 ng/ml recombinant CTGF and 100 ng/ml and 1 *μ*g/ml of Cyr61. Treatment with 10 ng/ml CTGF and treatment with 50 ng/ml significantly accelerated cell migration (Figure 7(c)). Treatment with either 100 ng/ml or 1 *μ*g/ml Cyr61 also accelerated cell migration at early time point (Figure 7(d)).

## 4. Discussion

During the past few years, plasma medicine has emerged as an important field in biomedical science. Cold atmospheric plasma generates a mixture of reactive oxygen and nitrogen species, including singlet oxygen, atomic oxygen, hydroxyl radicals, and hydrogen peroxide, as major (secondary) contributors. Further, especially long-living species can be generated upon contact with liquids [42, 43]. Various studies have focused on the anti-inflammatory, antimicrobial, antineoplastic, and wound healing promoting properties of CAP [13, 14, 44, 45]. In recent years, CAP has been used for the management of acute and chronic wounds in clinics; however, the mechanistic insight of CAP-mediated wound healing responses is still not investigated enough. Here, not only did we demonstrate that CAP enhances wound healing *in vitro*, but we also provided evidence regarding the CAP-mediated induction of differentially regulated genes and the paracrine effectors that promote wound healing via the HIPPO pathway.

Immortalized dermal keratinocytes and dermal fibroblasts were used for our present study, as these cells are primarily affected during skin/wound treatment. We also chose the *in vitro* keratinocyte-fibroblast coculture model for a portion of our studies, as this model provides the ability to examine the influence of fibroblasts on cocultured keratinocytes while omitting additional influences that are present in intact skin. For our experiments, we used the comparison of monocultured fibroblasts and keratinocytes on the one hand side with the coculture of both cell types, in order to identify possible paracrine interactions of those cells after treatment with CAP. These data are intended to support further research in chronic wound healing—mostly to compare data from diabetic patients. Therefore, it is important to understand how fibroblasts and keratinocytes influence each other to support wound healing after cold plasma treatment.

H_2_O_2_ is an important second messenger that is generated by CAP which triggers the release of growth factors during the wound healing process and has been shown to increase keratinocyte viability and migration in wound models [46]. Previous studies have revealed an increase in H_2_O_2_ in response to increasing CAP treatments [47]. Intracellular ROS levels also increase in keratinocytes after plasma treatment [48, 49]. High amounts of ROS/RNS species can affect human cells and lead to cell death and reduction in cell viability and proliferation [50]. Indeed, we observed that a 60 s CAP treatment significantly reduced cell viability in HaCaTs, GM Fbs, and cocultures within 3 hr. Cell viability in GM Fbs significantly decreased by greater than 50% after 24 hr, and based on this, we excluded the 60 s treatment from our subsequent experiments. However, short- and long-lived radicals also act as secondary messengers and contribute to wound healing processes [51]. Therefore, the aim of this study was to identify the stimulatory effects of CAP-generated reactive species on wound healing.

The epidermal layer functions as a barrier to the outside of the skin, and upon damage, newly divided keratinocytes migrate across the dermis [52]. Here, we optimized an *in vitro* coculture model that incorporated keratinocytes and fibroblasts. Unlike in single cell studies, this model for migration studies using coculture provides the opportunity to monitor the influence of each cell type on the other after CAP treatment. We observed that cells in coculture closely mimic real skin, and this system also displayed better CAP stimulation effects compared to those observed in the monocultures, indicating a crosstalk between the plasma-treated cell types. Many growth factors and cytokines are released from fibroblasts after injury, and this could provide an explanation for the acceleration of coculture wound closure [53–55].

To more thoroughly assess the paracrine signalling axis, we examined the signalling pathways involved in tissue homeostasis and regeneration. Emerging evidence suggests that the HIPPO pathway is regulated by a variety of signals, including mechanical stress, DNA damage, cellular stress, and oxidative stress [56]. Hence, we were interested in the HIPPO signalling pathway, as it plays an important role in cell growth, proliferation, and tissue homeostasis and is under redox control [25, 57]. YAP, a multifunctional adapter protein that possesses one coactivator domain, is a nuclear executer of the HIPPO signalling pathway. Based on our results, there was an increase in the mRNA expression of YAP in GM Fbs and HaCaT cells. We also detected a major upregulation of the YAP target genes CTGF and Cyr61 that was present primarily in GM Fbs and minimally in HaCaT cells. In accordance with this gene upregulation, CTGF and Cyr61 protein expression levels were also increased in GM Fbs. Upon phosphorylation of YAP, the HIPPO signalling cascade is blocked and phosphorylated YAP is retained in the cytoplasm. As YAP phosphorylation is increased after CAP treatment in HaCaT cells, this could provide a possible explanation for the minimal increase in CTGF protein and lack of Cyr61 detection in HaCaT cells; however, a significant decrease in YAP phosphorylation was not observed in GM Fbs. Therefore, it is likely that the interaction of cytoplasmic phosphorylated YAP with other signalling pathways (like Wnt, Notch, and TGF-*β*) can also contribute to the increased translation of CTGF and Cyr61 in GM Fbs [58–62]. Further studies are required to examine other upstream regulators that can induce CTGF and Cyr61 expressions.

The effect of NAC on restoring metabolic activity upon reduction of oxidative stress has been studied previously in other cell types [63]. In accordance with those results, in our immortalized skin cells, we also found that NAC restored and enhanced metabolic activity after reduction of CAP-mediated reactive species. This can be directly attributed to the ability of NAC to scavenge excess ROS within the media. CAP treatment can also reduce intracellular GSH levels in HaCaT cells as described previously by Dezest et al. [64]. As a cysteine-donating compound, NAC may act as a precursor to replenish the depleted cellular GSH upon CAP treatment, which in turn could induce the metabolic activity. Previous studies have also demonstrated the inhibitory effect of NAC on epithelial cell migration [65, 66]. Similar to these studies, we found that CAP-induced cell migration decreased by approximately 50% in response to 2.5 mM NAC treatment in HaCaT alone and in HaCaT cocultured with GM Fbs. Treatment with NAC reduced the mRNA expression of CTGF and Cyr61 in GM Fbs and Cyr61 in HaCaTs, and this confirms the effect of CAP in promoting the upregulation of this signalling pathway at the onset of wound healing. These findings are consistent with those of previous studies which observed that antioxidant treatment caused downregulation of CTGF and Cyr61 expressions in GO orbital fibroblasts and dermal primary fibroblasts, respectively [35, 36]. Therefore, we hypothesized that CAP could stimulate wound healing by triggering crosstalk between fibroblasts and keratinocytes that is mediated by HIPPO signalling.

Increasing numbers of studies are now focusing on the paracrine signalling and interaction between keratinocytes and fibroblasts in the context of wound repair [52, 67]. To test our hypothesis that CAP-induced stimulatory effects in coculture are a result of paracrine crosstalk, we monitored the cell migration of HaCaTs that were incubated with conditioned media harvested from CAP-treated GM Fbs (GM-Fb-CM) collected after 18 hr of CAP treatment. GM-Fb-CM was capable of accelerating HaCaT cell migration to a greater degree compared to that of CM harvested from untreated Fbs and no CM at all. This phenomenon supports our hypothesis that CAP induces paracrine signalling between these two cell types by releasing paracrine effectors and hence promoting keratinocyte migration. As expected, we could also detect an increase in secreted CTGF and Cyr61 expression in GM-Fb-CM treated with plasma. Additionally, HaCaT monocultures treated with recombinant CTGF and Cyr61 also exhibited improved cell migration. In particular, these data indicate that CTGF and Cyr61 secreted from CAP-treated fibroblasts act as paracrine effectors in wound healing. These results further confirm our hypothesis that CAP promotes a beneficial interaction between keratinocytes and fibroblasts that ultimately contributes to wound healing.

Here, for the first time, we demonstrated that CAP can stimulate a regenerative signalling pathway in dermal cells. We observed that the cold plasma-mediated effect on skin repair is mainly due to the activation of fibroblasts that when stimulated by CAP causes a paracrine stimulation of keratinocytes by releasing extracellular proteins as indicated by our coculture experiments. Together, these results confirm the role of the HIPPO pathway in plasma-mediated wound healing that is dominantly triggered by long-living ROS that are generated by cold plasma.

**Figure 8:**
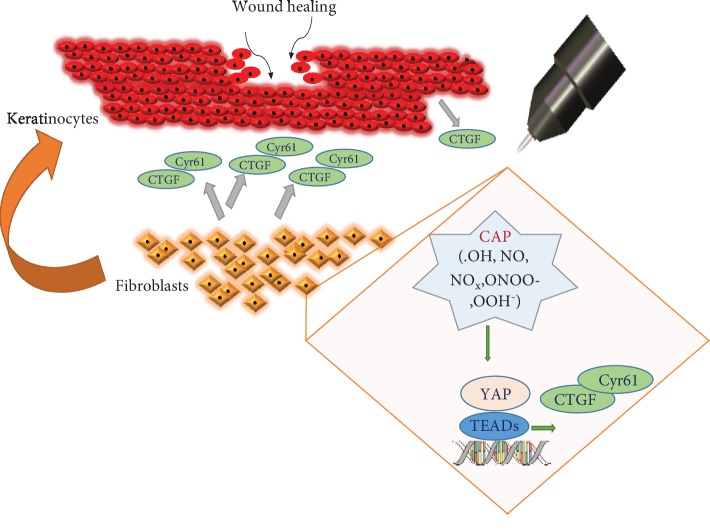
Schematic of keratinocyte activation by CAP modulated fibroblasts: the primary event in this scheme is the generation of reactive species in response to CAP treatment. These reactive species function as secondary messengers and stimulate the production of HIPPO signalling effectors such as CTGF in HaCaT and CTGF and Cyr61 by dermal fibroblasts in the vicinity. These extracellular matrix proteins that are released from fibroblasts in turn activate keratinocytes, accelerate migration, and promote wound healing.

An overview of the impact of CAP treatment on the cellular redox signalling based on the present data is provided in Figure 8.

## 5. Conclusions

CAP treatment results into complex crosstalk between redox signalling pathways in mammalian cells via the impact of short- and long-lived redox species. kINPen Med has already received approval for clinical applications in wound healing [12, 68]. Here, we revealed a mechanistic insight into wound healing where CAP induced a YAP-CTGF-Cyr61 signalling axis in dermal fibroblasts that was inhibited by antioxidant treatment. Although dermal keratinocytes could minimally produce HIPPO downstream effector CTGF, it is the secretion of CTGF and Cyr61 from fibroblasts that influences the activation of keratinocytes through paracrine signalling and improves cell migration upon wound onset.

## Data Availability

The majority of the data can be found in the manuscript, and further data used to support the findings of this study are available from the corresponding authors upon request.

## Abbreviations

CAP: Cold atmospheric plasma
CM: Conditioned medium
CTGF: Connective Tissue Growth Factor
Cyr61: Cysteine-rich angiogenic protein 61
YAP: Yes-associated protein 1
TEAD: TEA domain family member
GM Fbs: GM00637 fibroblasts
NAC: N-Acetyl L cysteine
TGF-*β*: Transforming growth factor *β*
RNS: Reactive nitrogen species
ROS: Reactive oxygen species.

## Supplementary Materials

Supplementary Materials Figure S1: summary of cell count in monoculture and coculture. Figure S2: forward and reverse primer sequences used in real-time PCR for YAP, Cyr61, and CTGF. Figure S3: Amplex Red assay was performed to quantitatively measure the production of H_2_O_2_ in response to CAP treatment. Figure S4: wound healing images at times 0, 12, and 24 hr in untreated and CAP-treated coculture. Figure S5: wound healing images at times 0, 12, and 24 hr in untreated and CAP-treated HaCaT cells. Figure S6: wound healing images at times 0, 12, and 24 hr in untreated and CAP-treated GM Fbs.
