# Supplementary material for: The HIPPO Transducer YAP and Its Targets CTGF and Cyr61 Drive a Paracrine Signalling in Cold Atmospheric Plasma-Mediated Wound Healing
Source: Oxid Med Cell Longev. 2020 Feb 13;2020:4910280. doi: 10.1155/2020/4910280 (PMC7040405; doi:10.1155/2020/4910280)

Fig S1: Summary of monoculture and co-culture

Monoculture	GM Fbs	HaCaT	Time of the assay	Plate type
**In vitro scratch assay**	6 x 10^4	6 x 10^4	24 hr	96 well Image lock plate
**In vitro scratch assay (HaCaT monoculture stimulated with CTGF, Cyr61 or GM Fb CM)**	-	4 X 10^4	24hr	96 well Image lock plate
**Metabolic Activity assay**	2.1 X 10^4	2.1 X 10^4	3hr, 24hr	96 well plate
**Harvest of GM Fb CM**	0.1 X 10^6	-	18hr	24 well plate

Co-culture	GM Fbs	HaCaT	Time of the assay	Plate type
**In vitro scratch assay**	2 X 10^4	4 X 10^4	24 hr	96 well Image lock plate
**Metabolic Activity assay**	0.7 X 10^4	1.4 X 10^4	3hr, 24hr	96 well plate

FigS2: Forward and reverse primer sequences used in Real time PCR for YAP, Cyr61 and CTGF

YAP fwd	TAGCCCTGCGTAGCCAGTTA
YAP rev	TCATGCTTAGTCCACTGTCTGT
Cyr61 fwd	GGTCAAAGTTACCGGGCAGT
Cyr61 rev	GGAGGCATCGAATCCCAGC
CTGF fwd	CAGCATGGACGTTCGTCTG
CTGF rev	AACCACGGTTTGGTCCTTGG

Fig S3: Amplex Red assay to monitor H_2O_2 production

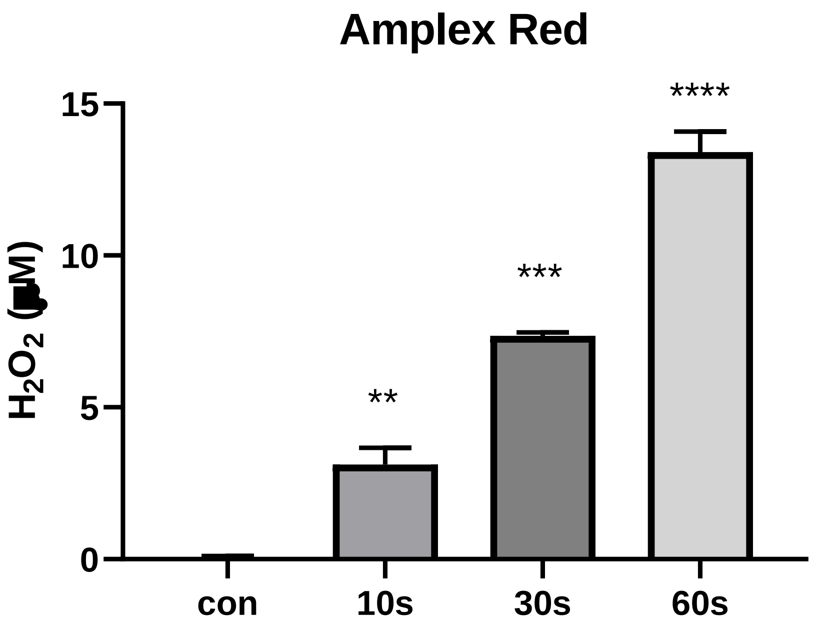

FigS4: Wound healing images at times 0, 12 and 24 hour in untreated and CAP treated co-culture

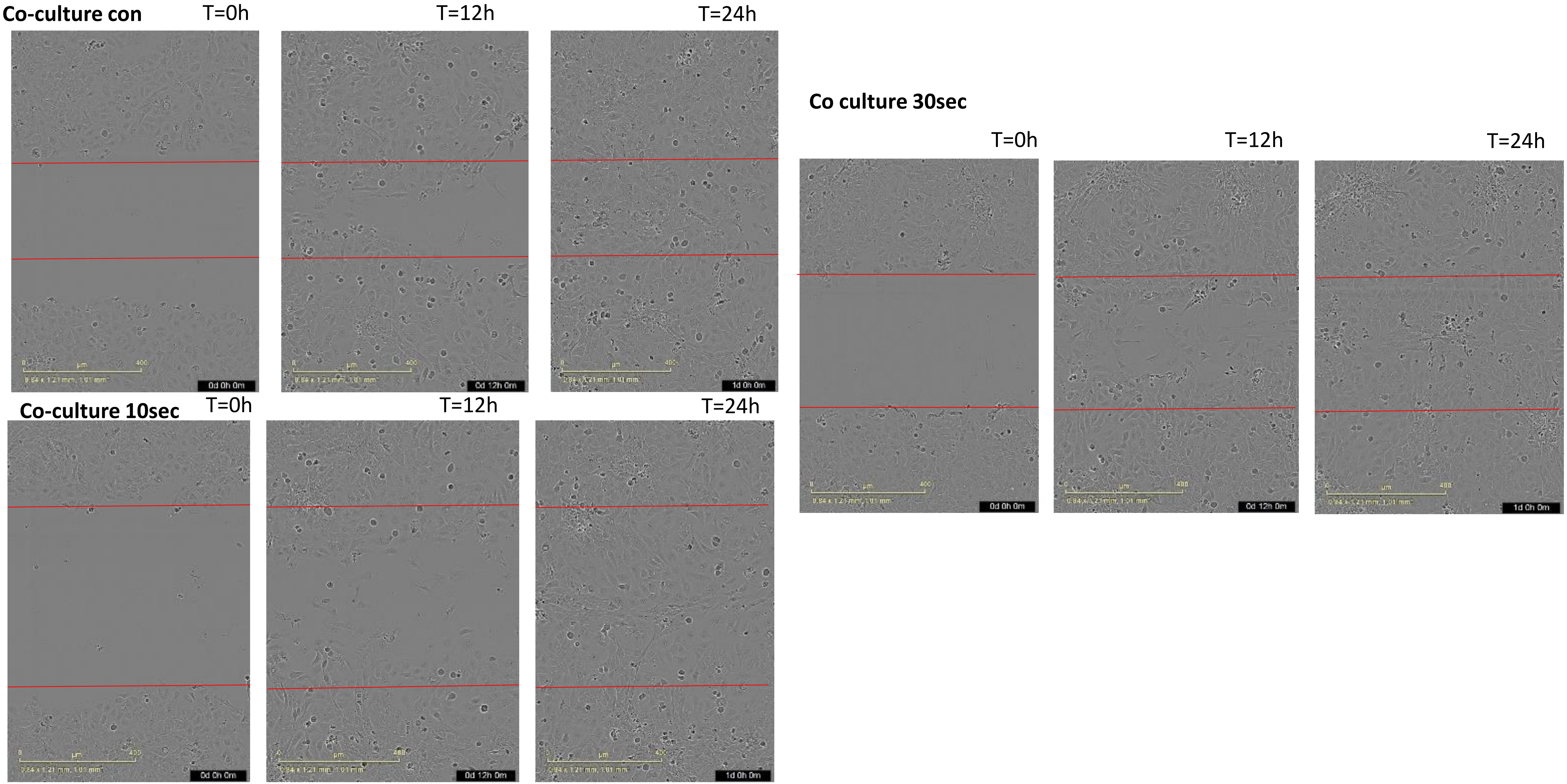

FigS5: Wound healing images at times 0, 12 and 24 hour in untreated and CAP treated HaCaT cells

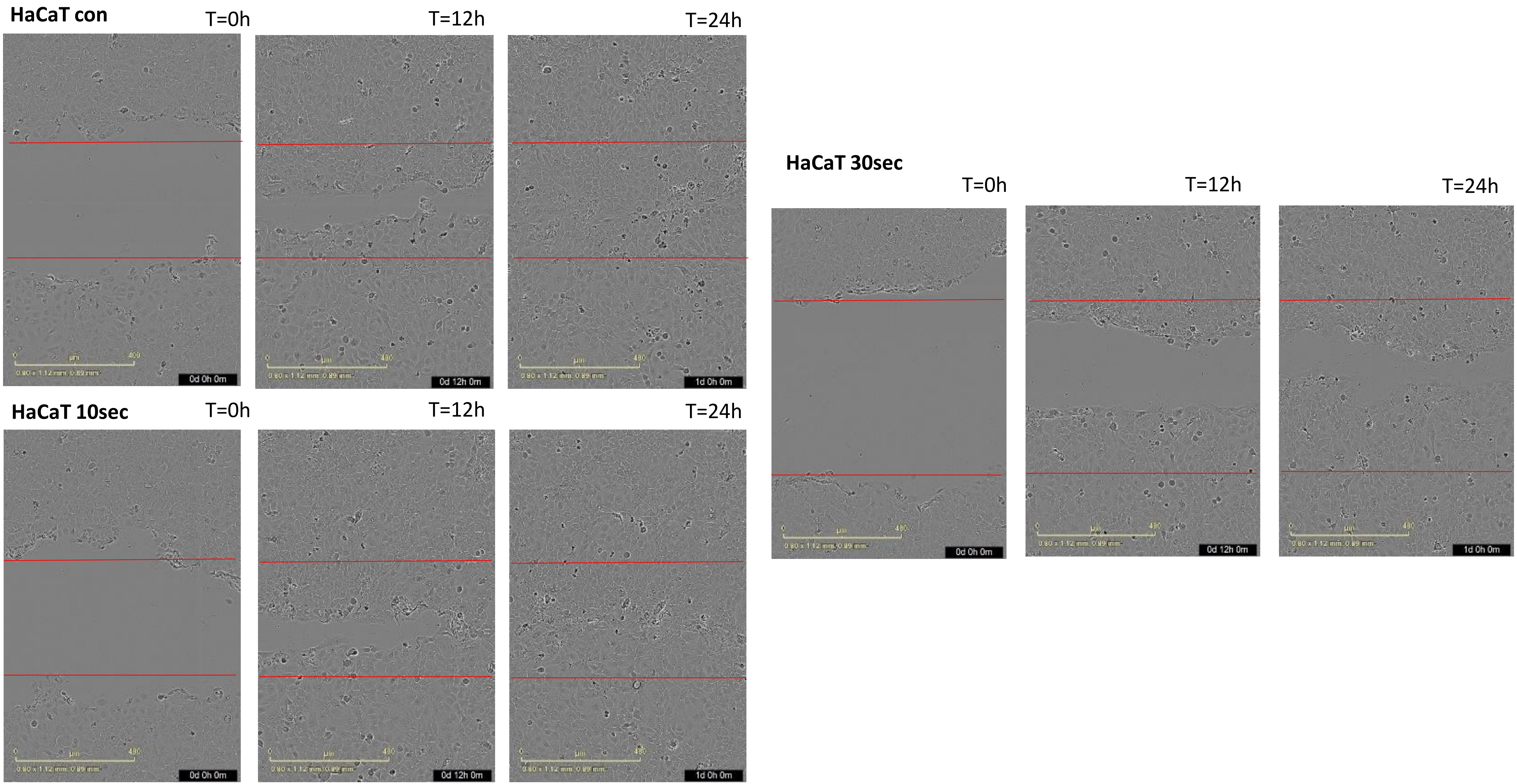

FigS6: Wound healing images at times 0, 12 and 24 hour in untreated and CAP treated GM Fbs

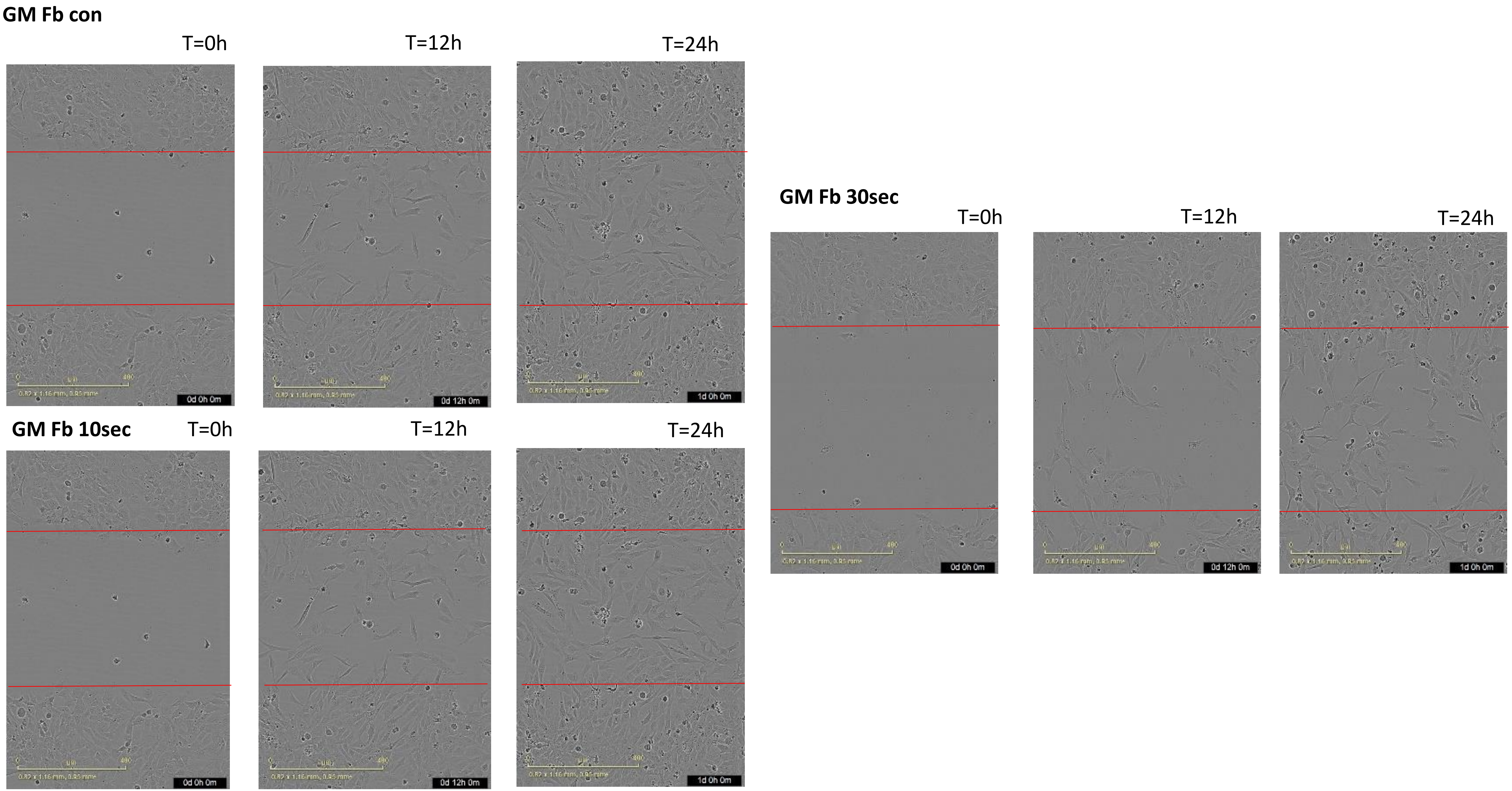

Supplement: Supplementary Materials — Figure S1: summary of cell count in monoculture and coculture. Figure S2: forward and reverse primer sequences used in real-time PCR for YAP, Cyr61, and CTGF. Figure S3: Amplex Red assay was performed to quantitatively measure the production of H2O2 in response to CAP treatment. Figure S4: wound healing images at times 0, 12, and 24 hr in untreated and CAP-treated coculture. Figure S5: wound healing images at times 0, 12, and 24 hr in untreated and CAP-treated HaCaT cells. Figure S6: wound healing images at times 0, 12, and 24 hr in untreated and CAP-treated GM Fbs. [file 4910280.f1.pdf]
